# Functional Zoning and Path Selection of Land Comprehensive Consolidation Based on Grey Constellation Clustering: A Case Study of Dongying City, China

**DOI:** 10.3390/ijerph19116407

**Published:** 2022-05-25

**Authors:** Yaoben Lin, Danling Chen

**Affiliations:** 1 School of Law and Public Affairs, Nanjing Tech University, Nanjing 211816, China; lyb@njtech.edu.cn; 2 Nanjing Institute of Geography and Limnology, Chinese Academy of Sciences, Nanjing 210008, China; 3 Department of Land Management, College of Public Administration, Huazhong Agricultural University, Wuhan 430070, China

**Keywords:** land comprehensive consolidation, GIS analysis, grey constellation clustering, functional zoning, path selection

## Abstract

The functional zoning of land comprehensive consolidation and the selection of consolidation paths are the key content of the current land and space planning, and it is also an important measure to achieve regional sustainable development. At present, the research system on land comprehensive consolidation is not yet mature. The previous research area is relatively small and not representative, and an effective method system has not been formed. Research on the selection of functional zoning and a consolidation path is also relatively scarce. There is an urgent need to construct the theory and method system of land comprehensive consolidation functional zoning and consolidation path selection. Taking Dongying City in China as an example, this paper constructs a zoning index system from four aspects including natural conditions, location advantages, social economy and land use. The entropy method is used to determine the weights, and GIS spatial data visualization is used to analyze the spatial distribution characteristics of the index system. Based on the analysis, the grey constellation clustering method is used to divide the study area into four types of land comprehensive consolidation functional areas, and the results of functional zoning are adjusted according to the ranking of comprehensive index values, the principle of maximum similarity, and the continuity of natural space. The research results show that: ① There are 10 functional zonings for urban development and ecological protection land consolidation, and the main consolidation path should be the ecological country park consolidation model, which can effectively serve the urban ecological construction. ② There are nine functional zonings for rural development and cultivated land conservation consolidation, and their consolidation path should be based on a comprehensive rural improvement model that enhances the quality of the village and the development, utilization and protection of cultivated land resources. ③ There are 18 functional zonings for cultivated land improvement and ecological protection land consolidation, and the main consolidation path should focus on the cultivated land ecological improvement mode that emphasizes the quality of cultivated land and the improvement of regional ecological functions. ④ There are four functional zonings for ecological conservation and fallow recuperative land consolidation, and their main consolidation path should be the land ecological restoration and improvement model of construction land reclamation, cultivated land ecological conservation, and conversion of farmland to forest and grassland. The research results can provide references for Dongying City to formulate land and space planning, and can be extended to the design of comprehensive land remediation projects in other regions. It is of great significance to promote regional sustainable and scientific development.

## 1. Introduction

As an important place to carry human life and production, land plays a significant role in promoting social development and human progress. In addition, land is also complex, with social and natural attributes. While bearing the pressure of human survival and development, it also bears the task of ecological environmental protection and construction [1,2]. Therefore, the rational use of land is of great significance to the development of human society and the protection of the earth’s environment [3]. However, as the population continues to grow, urban construction land continues to sprawl, occupying a large amount of natural ecological land, which has caused urban heat islands, ecological deterioration, and climate change problems [4,5]. As an effective means to solve the problem of land use, land consolidation has always received extensive attention from scholars all over the world [6,7,8]. Land consolidation is an activity of artificially transforming land resources. It uses engineering construction measures to comprehensively renovate the land to improve land-use efficiency, production, and living and ecological environment conditions [9,10].

Reviewing the characteristics of global land consolidation development, it can be seen that the initial land consolidation was mainly to solve the problem of serious fragmentation of cultivated land and the restriction of agricultural development [11,12]. Through land consolidation, it is possible to dredge the obstacles caused by the mismatch of water, land, manpower, mechanization and other production factors [13]. Population growth requires more farmland to sustain food production, while urbanization will lead to land fragmentation, which greatly increases the demand for cultivated land protection [14]. In rural areas, the fragmentation of cultivated land has had a great negative impact on capital investment, sustainable economic and social development, and ecological and environmental protection [15]. A scientific and reasonable land consolidation plan can effectively solve the problem of arable land fragmentation [16]. It is an agricultural development policy that can increase arable land intensification, increase crop output, and improve the ecological environment of farmland [17]. It is bound to become a necessary measure for sustainable development in rural areas [18,19]. The functional zoning of land comprehensive consolidation can effectively solve the problem of rural land use, realize the sustainability of land use to ensure food security, and improve the spatial structure of the land by building roads, irrigation facilities and other infrastructure [20,21].

As a complex system project, land comprehensive consolidation also needs to consider the wishes and demands of stakeholders [22,23]. Therefore, land consolidation usually has multi-functional characteristics to meet the various needs of social development [24]. As the demand for regional development continues to change, people are gradually paying attention to the improvement of the quality of life, which puts forward higher requirements for land consolidation. After entering the 21st century, traditional land consolidation measures such as merging land, leveling land, and building roads and ditches are no longer the focus of research. Now, the international community pays more attention to the research on new areas of land consolidation such as landscape design, environmental management and water and soil protection [25]. Many countries and regions mainly focus on water and soil erosion, landscape function decline, land degradation and other issues [26,27]. Based on the research on farmland protection, water and soil conservation and landscape restoration technologies and their ecological environmental effects emphasize the overall promotion of various projects in the land consolidation area [28,29], regarding ecological consolidation as a necessary means to maintain the sustainable development of the consolidation area, while emphasizing the accumulation and progress of multiple goals in land consolidation, such as ecological remediation, natural landscape design, cultural landscape protection and inheritance [30,31,32]. It can be seen that land consolidation has become a modern practical application with multi-objective and multi-functional characteristics, which is of great significance to the realization of sustainable agriculture, rural and urban development and ecological environment protection [33,34]. Therefore, the functional zoning of land consolidation is a necessary measure to meet the developmental needs of the times.

The functional zoning of land comprehensive consolidation is an effective reference result for land and space planning, and it is also the basis for the implementation of land consolidation planning [35]. It provides scientific guidance for the deployment of land consolidation projects, speeding up the restoration of damaged land and optimizing the land-use structure, and is of great significance for determining the key direction of land consolidation and carrying out differentiated land consolidation. At present, there are few systematic studies on the functional zoning of land comprehensive consolidation, most of which are based on physical geography, and lack comprehensive consideration of regional socio-economics [31,36]. In addition, most research only carries out the zoning work of land consolidation from the single land-use types such as rural residential areas, ecological land and arable land, and there is much less research on the functional zoning of comprehensive land consolidation in a large area [29,37,38]. Internationally, the functional zoning of land comprehensive consolidation is mainly carried out through quantitative and qualitative methods. Expert consultation and public discussion are the most commonly used qualitative methods, which can effectively reflect the regional characteristics of land consolidation, but they are subjective and one-sided [28,39]. The index system method and the principal component analysis method are quantitative analysis methods that are commonly used in land consolidation zoning, but they can only reflect the difference in regional values and cannot reflect the actual situation [31]. In addition, with the development of the times, the connotation of land consolidation has also undergone tremendous changes, and the original zoning methods and concepts can no longer meet the existing land comprehensive consolidation needs. In order to highlight the functionality, scientificity and effectiveness of land comprehensive consolidation zoning, the construction of the conceptual framework and method system of land comprehensive consolidation functional zoning is particularly important.

In view of this, this study takes China’s Dongying City as the research area and uses townships as the basic research unit to conduct a more detailed classification study. Starting from the functional requirement orientation, using methods such as GIS analysis, the entropy method and the grey constellation clustering method to analyze the indicators required by the various functions required for regional development, so as to form a system of identification indicators for each function to implement the functional zoning of land comprehensive consolidation. According to the results of functional zoning, the positioning of land consolidation functional zoning is established, and the focus of each land comprehensive consolidation functional zoning is determined.

## 2. Methodology

### 2.1. Study Area and Data Source

**Figure 1:**
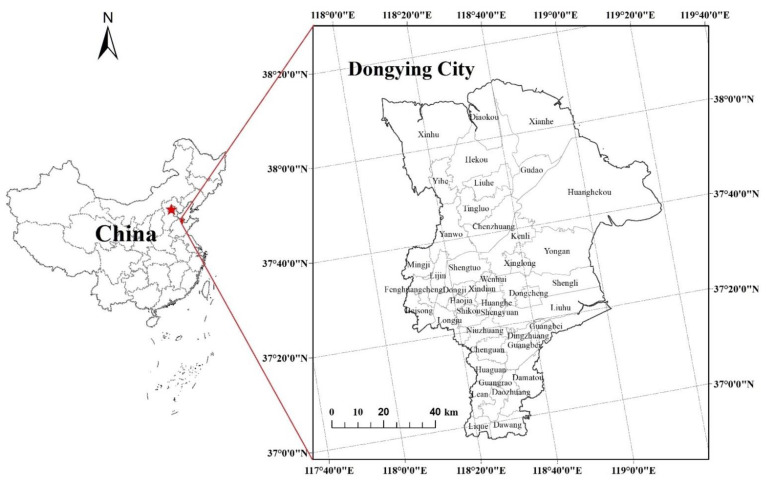
Geographical location of Dongying.

Dongying City, the study area, is located in the eastern coastal area of China (118°5′ east longitude, 38°15′ north latitude). Under the joint influence of the Eurasian continent and the western Pacific, it has a warm temperate continental monsoon climate. The basic climate features are cold in winter and hot in summer, with four distinct seasons. The multi-year average temperature is 12.8 ℃, the frost-free period is 206 days, and the accumulated temperature of not less than 10 ℃ is about 4300 ℃, which can meet the crops’ three crops in two years. The average annual precipitation is 555.9 mm, mostly concentrated in summer, accounting for 65% of the annual precipitation. The inter-annual variation of precipitation is large, which is prone to drought and waterlogging disasters. The rise of Shengli Oilfield, the city’s second-largest oil industry base in China, has brought huge benefits to the local people, and its per capita GDP ranks among the top in China. In addition, Dongying City is located at the mouth of the Yellow River and has beautiful landscapes. It was named one of China’s “six most beautiful wetlands”. However, in recent years, the contradiction between social development and ecological environment protection has deepened. Therefore, it is extremely urgent and necessary to carry out the division of land comprehensive consolidation functions. Therefore, this study selects China’s Dongying City as the research area because the city is located in the Yellow River Delta, with a developed economy but a fragile ecological environment, which is representative of the research. The geographical location of Dongying City is shown in Figure 1.

The land-use data of cultivated land, forest land, construction land, rivers and roads used in this article come from the 2020 survey data provided by the Dongying Natural Resources Bureau. Demographic data and socio-economic data come from the statistics of Dongying City Statistics Bureau. The topographic undulations come from DEM data with a resolution of 30 m in Dongying City downloaded from the National Earth System Science Data Center, and the newly-added cultivated land area comes from statistical data provided by the Dongying City Land Consolidation Center.

### 2.2. Analysis Methods

#### 2.2.1. Construction of Evaluation Index System of Land Renovation District

**Figure 2:**
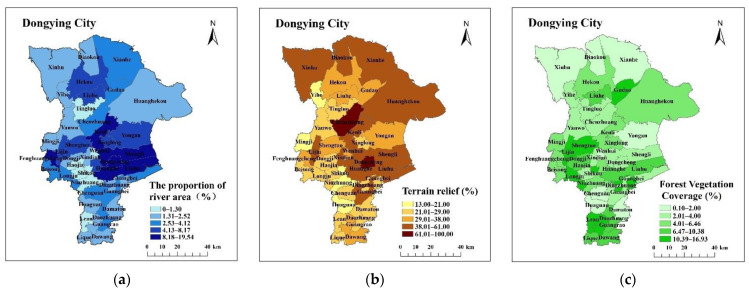

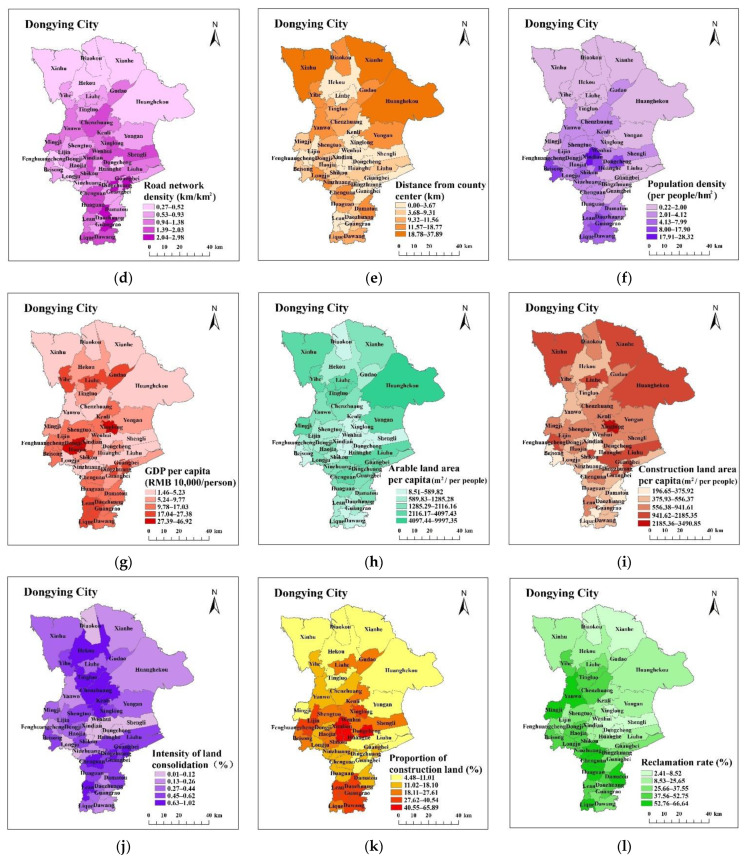
Spatial distribution of each index: (**a**) The proportion of river area; (**b**) Terrain relief; (**c**) Forest Vegetation Coverage; (**d**) Road network density; (**e**) Distance from county center; (**f**) Population density; (**g**) GDP per capita; (**h**) Arable land area per capita; (**i**) Construction land area per capita; (**j**) Intensity of land consolidation (**k**) Proportion of construction land; (**l**) Reclamation rate.

The functional zoning of land comprehensive consolidation will be affected by many factors in the study area, including topography and landforms, cultivated land quality, traffic accessibility, basic supporting facilities, irrigation conditions, and socio-economic conditions. When selecting indicator factors, it is necessary to consider the comprehensive, hierarchical and regional principles of indicator selection, which will not only affect the construction of the indicator system but also reflect the interaction of indicators [19]. Taking into account the experience of selecting the index system of land consolidation function zoning, the basic data of Dongying city and the principle of selecting the index, the paper establishes 12 index systems of land comprehensive consolidation function zoning, which consists of four aspects: natural attribute, location attribute, social-economic attribute and land-use attribute. See Table 1 and Figure 2 for details.

Natural attributes mainly include terrain relief, forest vegetation coverage and the proportion of river area. The topographic relief reflects the topography, elevation and altitude of each township, affects the distribution trend and land-use pattern of cultivated land, residential sites, and construction land, and also determines the difficulty of land consolidation. The greater the terrain relief, the more difficult the land consolidation work and the greater the investment. The coverage of forest vegetation is an indirect reflection of the construction of a regional ecological environment, and the land consolidation work should develop in harmony with the ecological environment. The greater the forest vegetation coverage, the greater the proportion of forest land, and the more important the ecological protection function it carries. Therefore, the focus of land consolidation in areas with greater forest vegetation coverage should be to protect forest land and ensure soil erosion capacity. The proportion of river area is an important guarantee for the irrigation capacity of the region, and it affects the irrigation capacity required by the region to a certain extent. Regional irrigation conditions with a large river area ratio are relatively superior, which is more favorable to the development of agriculture and provides convenient conditions for the construction of high-standard basic farmland.

The location attribute plays a guiding and restraining role in the distribution of cultivated land area, population and settlements. The location factor includes two indicators: distance from Dongying urban area and road network density. The distance from the Dongying urban area can indirectly reflect the main development trend of land-use patterns. The closer the distance between the township and the Dongying urban area, the closer to the city center, the greater the proportion of construction land, the greater the possibility of agricultural land non-agricultural. The density of road networks directly reflects the convenience of traffic roads in towns and cities, and it is also an indirect reflection of the difficulty of land consolidation. The denser the road network, the easier the land consolidation work.

The socio-economic attributes guide the regional land-use pattern and the direction of land consolidation, which are mainly represented by four indicators: population density, per capita GDP, per capita cultivated land and per capita construction land. Population density reflects the degree of intensive and economical use of land in each township. GDP per capita reflects the economic development level of each township. The economic development of Dongying City is mainly based on the primary and secondary industries, and the per capita GDP can also indirectly reflect the level of arable land output. The per capita cultivated land reflects the comparative relationship between the population and cultivated land, which is used to illustrate the scarcity of cultivated land resources. The construction land occupied per capita reflects the land-use situation of each township residential area. The population density is large and the per capita GDP is small, indicating that the land consolidation direction is urban. The larger the proportion of per capita arable land and the smaller the proportion of per capita construction land, the direction of land consolidation is to focus on high-standard basic farmland construction.

Land-use attributes are mainly used to measure the land utilization rate and the intensity of land consolidation and are represented by three factors: the intensity of land consolidation, the proportion of construction land, and the reclamation rate. The reclamation rate reflects the land-use suitability, current situation and development and utilization trend of cultivated land. The proportion of construction land reflects the overall pattern of land use in each township. The larger the proportion, the greater the population, so it should be developed into an urban-type, and land consolidation should focus on intensive land conservation. The intensity of land consolidation reflects the potential of the amount of agricultural land consolidation in each township, indicating the size of the land consolidation space in the township.

#### 2.2.2. Determination of Indicator Weights and Constellation Cluster Analysis

##### Calculation of Indicator Weights

Entropy is a physical quantity, first proposed by the German physicist Rudolf Clausius in 1850, to express the uniformity of the distribution of any kind of energy in space [40]. The more uniform the energy distribution, the greater the entropy. According to the characteristics of entropy, the randomness and disorder degree of an event can be judged by calculating the entropy value, and the discrete degree of an indicator can also be judged by the entropy value. The greater the degree of dispersion of the index, the greater the impact (weight) of the index on the comprehensive evaluation, and the smaller the entropy value. The specific steps are as follows.

(1) Data normalization

Due to the different meanings represented by each index, there are differences in dimensions. In order to eliminate this difference, each indicator needs to be dimensionless. This is normalization.

Positive indicators:

(1) $$X_{ij}^{\prime }=\frac{X_{ij}-X_{j\min}}{X_{j\max}-X_{j\min}}$$

Negative indicators:

(2) $$X_{ij}^{\prime }=\frac{X_{j\max}-X_{ij}}{X_{j\max}-X_{j\min}}$$

Among them, $X_{ij}^{\prime }$ represents the normalized value of the *j*th index of the *i*th township, $X_{ij}$ represents the actual value of the *j*th index of the *i*th township, $X_{j\min}$ and $X_{j\max}$ are the minimum and maximum values of the *j*th index of the townships in the study, *i* = 1, 2,..., *n* and *j* = 1, 2,..., *m*.

(2) Calculate the proportion *P_ij_* of the *i*th township to the index of the *j*th index

(3) $$P_{ij}=\frac{X_{ij}^{\prime }}{\sum_{i=1}^{n}X_{ij}^{\prime }}$$

(3) Calculate the entropy value *e_j_* of the *j*th index

(4) $$e_{j}=-k\sum_{i=1}^{n}P_{ij}\ln P_{ij}=-\frac{1}{\ln n}\sum_{i=1}^{n}P_{ij}\ln P_{ij}$$

Among them, *k* = 1/ln(*n*) > 0, satisfying *e_j_* ≥ 0

(4) Calculate the information entropy redundancy $d_{j}$

(5) $$d_{j}=1-e_{j}$$

(5) Calculate the weight $W_{j}$ of each indicator

(6) $$W_{j}=\frac{d_{j}}{\sum_{j=1}^{m}d_{j}}$$

The weight calculated by the entropy method is completely based on conceptual principles and mathematical formulas and has strong objectivity, but it often lacks the subjective intention of decision-makers and lacks pertinence. Therefore, based on the entropy method to determine the weights, this paper invites relevant experts to rank the importance of each index in the index system, merges and rectifies the evaluation results of all experts, and finally adjusts individual indexes. Through the combination of qualitative and quantitative, the final indicator weight is determined.

The weight of each indicator is shown in Table 2.

##### Constellation Cluster Analysis

The basic principle of grey constellation clustering is to place each sample point in a semicircle according to a certain quantitative relationship, each sample point is represented by a star point, and the same kind of sample points can form a “constellation” [41,42]. Then, they are categorized and the boundaries of different “constellations” are distinguished, resulting in a constellation type diagram. Simply put, it is to first convert each index value into an angle value and then multiply each angle value by the weight of each index to determine its importance in the index system. Then, the angle value is converted into a coordinate value through trigonometric function, and finally, the calculated coordinate value falls into the semicircle of 0–180°, reflecting the similarity and aggregation degree of each sample point. Samples with a high degree of aggregation or similarity are grouped together and called a “constellation”. The specific steps are as follows:

(1) Data transformation

(7) $$\Phi_{ij}=\frac{X_{ij}-X_{j}{}_{{}_{\min}}}{X_{j}{}_{{}_{\max}}-X_{j}{}_{{}_{\min}}}\times 180{}^{\circ}$$

(8) $$\Phi_{ij}=\frac{X_{j}{}_{{}_{\max}}-X_{ij}}{X_{j}{}_{{}_{\max}}-X_{j}{}_{{}_{\min}}}\times 180{}^{\circ}$$

In the formula: *i* = 1, 2,..., *n*, *i* is the number of sample points (here represents each township); *j* = 1, 2,..., *m*, *j* is the number of indicators (here represents each categorical variable); $\Phi_{ij}$: transformed data; $X_{ij}$: original data; $X_{j}{}_{{}_{\max}}$: maximum value of the *j*th variable; $X_{j}{}_{{}_{\min}}$: minimum value of the *j*th variable.

(2) Assign value to the selected indicator

Calculate the weight *w_j_* of each index by using the entropy method of Formulas (1)–(6), so that: 0 ≤ *w_j_* ≤ 1, and $\sum_{j=1}^{m}w_{j}=1$ (*j* = 1, 2,..., *m*), *m* is the number of indexes. The weight of each indicator shown in Table 2.

(3) Calculate the coordinate value of each sample point (township)

(9) $$x_{i}=\sum_{j=1}^{m}w_{j}\cos\Phi_{ij}$$

(10) $$y_{i}=\sum_{j=1}^{m}w_{j}\sin\Phi_{ij}$$

In the formula: *i* = 1, 2,..., *n*, *i* is the number of sample points (township); *j* = 1, 2,..., *m*, and *j* is the number of indicators; *x_i_* is the abscissa of the *i*-th sample point (township); *y_i_* is the ordinate of the *i*-th sample point (township); *w_j_* is the weight of the *j*-th index.

(4) Draw a constellation diagram

According to the values of *X_i_* and *Y_i_*, the position of each sample point is determined, and the representative star points of the sample points with similar and close properties are gathered together to form a “constellation diagram”.

(5) Calculate the comprehensive index value

(11) $$z_{i}=\sum_{j=1}^{m}\Phi_{ij}w_{j}$$

In the formula, *z_i_* is the comprehensive index value and *w_j_* is the index weight.

Calculate the comprehensive index value of each sample point according to Formula (11), and then sort all the sample points to test the clustering result.

## 3. Results and Discussion

### 3.1. Functional Zoning Results of Comprehensive Land Consolidation

Traditional land consolidation zoning methods are generally based on qualitative and comprehensive analysis and are spatially clustered and connected, with fewer occurrences of separation and enclaves. In this paper, the quantitative partitioning method of index system and evaluation combined with constellation clustering is adopted. Therefore, the partition results may have the characteristics of fragmentation and spatial discontinuity in space.

**Figure 3:**
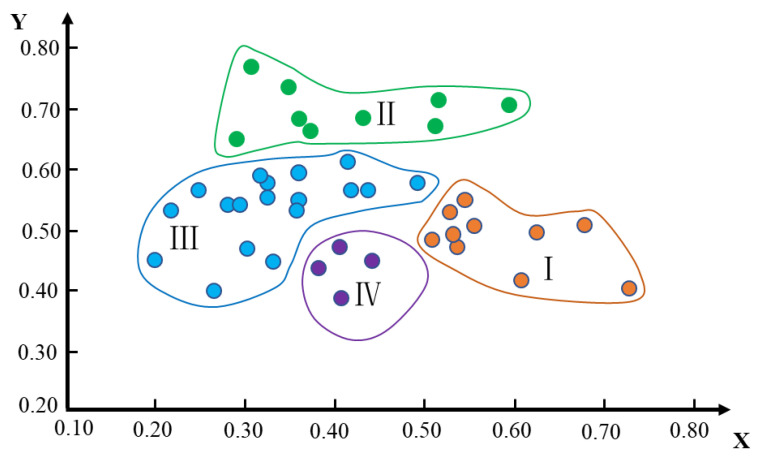
Grey constellation clustering results. Note: I, II, III, and IV are urban development and ecological protection type, urban development and cultivated land protection type, cultivated land improvement and ecological protection type, ecological conservation and fallow cultivation type, respectively.

This study fully considers the principles of sustainable development, consistency with natural landform units, ecological protection, protection of basic farmland, food security, and local conditions. Using the grey constellation clustering method, the coordinate points of 41 townships were calculated. Then, these coordinate points were drawn on the constellation map, and according to the characteristics of these coordinate points, the study area could then be divided into four types of land comprehensive consolidation areas, as shown in Figure 3.

### 3.2. Adjustment and Verification of Clustering Results

The functional zoning method of land comprehensive consolidation used in this paper is to sort 41 townships according to the comprehensive scores calculated by the grey constellation clustering method. Considering the principle of maximum similarity and the natural spatial continuity of land comprehensive consolidation, combined with the spatial correlation and difference of natural attributes, location attributes, socio-economic attributes and land-use attributes of Dongying City. The clustering results are appropriately merged and adjusted to facilitate the implementation, planning and unified governance of the land comprehensive consolidation plan in the later stage. The results after adjustment are compared with the results before adjustment and the changes are small, indicating that the grey constellation clustering method is feasible for the functional zoning results of land comprehensive consolidation in Dongying City.

### 3.3. Clustering Results and Consolidation Path Selection

**Figure 4:**
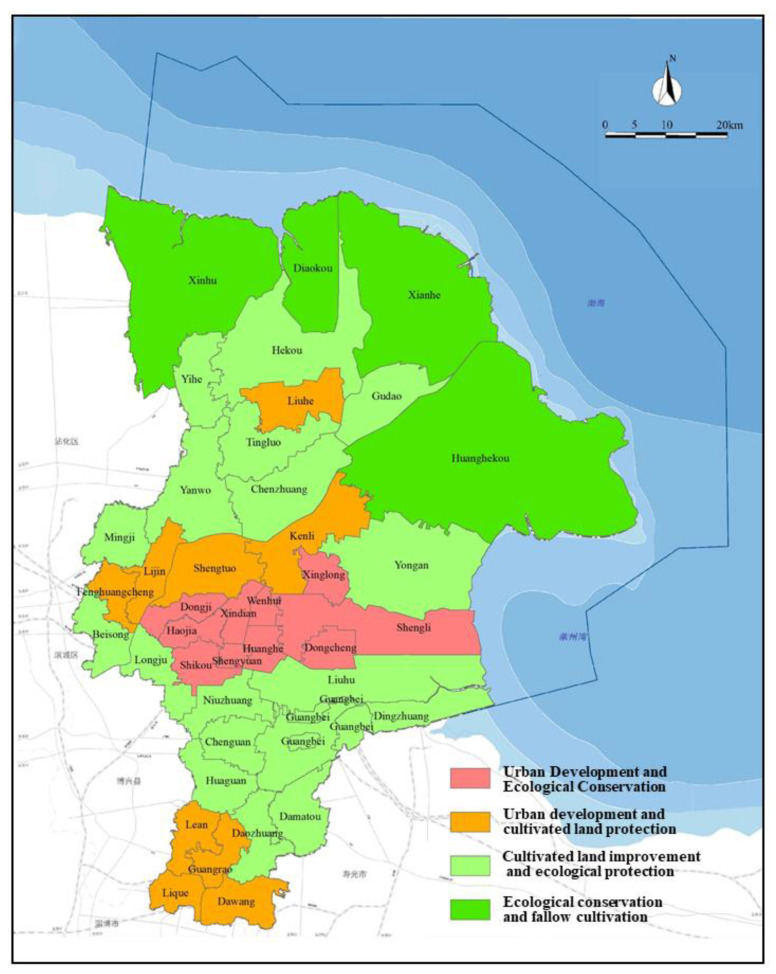
Functional zoning map of land comprehensive consolidation in Dongying City.

According to the above adjustment results, the functional zoning map of land comprehensive consolidation in Dongying City is obtained, as shown in Figure 4.

(1) Urban development and ecological protection land comprehensive consolidation functional area. The functional area includes 10 towns and towns including Dongcheng Street, Shengli Street and Xinglong Street, covering an area of 825.39 km^2^, accounting for about 10% of the total area. This area belongs to the plain area with gentle terrain and is concentrated in the center of Dongying City. This area has the most developed road network, the highest population density and the highest proportion of construction land among the four functional areas of Dongying City. This area should combine the needs of urban development, learn from the construction of country parks in European countries, organically combine the expansion of urban construction land with the ecological improvement of land, and explore the coordination and unity of land and urban development [43], Moreover, it should carry out an urbanization-led land consolidation model, carry out surveys and consolidation activities on the status of urban land use such as unused land and hollow villages, and coordinate urban and rural development and intensive use of land resources.

(2) Urban development and cultivated land conservation land comprehensive consolidation functional area. The functional area includes nine townships including Lijin Street, Fenghuangcheng Street and Dawang Town, with an area of about 1052.40 km^2^, accounting for 12.76% of the total research area. This area is distributed in the central, southern and northern parts of Dongying City, and is an area with close proximity to the county seat, convenient transportation and better economic development. In addition, the area has a reclamation rate of 36.4%, which is higher than the city’s average and has a large area of arable land. This area can make full use of land resources such as abandoned land in townships and towns, idle land and cultivated land occupied by roads through land comprehensive consolidation methods such as relocation of villages and sites, and internal potential digging. This can effectively improve the level of intensification and conservation of the spatial pattern of land use in townships, increase the reclamation rate, and improve the phenomenon of hollow villages and idle land. As in Slovenia, rural agriculture and economic revitalization are achieved through land comprehensive consolidation [44]. Therefore, while developing urbanization, this region should strengthen the protection of cultivated land and utilize convenient transportation conditions and cultivated land resources to develop agriculture, so as to promote the coordinated development of urban and rural areas.

(3) Cultivated land improvement and ecological protection land comprehensive consolidation functional area. There are 18 townships including Beisong Town, Yanwo Town, Chenzhuang Town, etc., with an area of about 3402.54 km^2^, accounting for 41.26% of the total research area. The topographic relief of this area is small, the terrain is gentle, and it belongs to the plain area. The reclamation rate in this area is the highest, and the per capita GDP is also at a relatively high level. High-standard basic farmland, mainly Guangbei farms, are also located in this area. There are many towns in this region with a reclamation rate as high as about 60%, and the output capacity of arable land is high. Land comprehensive consolidation in this region should focus on increasing the quantity of arable land and improving the quality of arable land. It is possible to optimize the agricultural production pattern through field consolidation, enhance the basic farmland supporting irrigation facilities, and improve the ability of farmland infrastructure to serve agricultural production. These areas can refer to Japan’s cultivated land consolidation model to achieve the coordinated development of agriculture and the environment [45]. This will help improve the comprehensive economic benefits of agricultural land, accelerate the development of modern agriculture, and build high-standard basic farmland. At the same time, the forest coverage rate and river area in this region are relatively large. While developing agriculture, we should also pay attention to the conservation of the ecological environment, so as to realize the sustainable development of the regional economy and ecology.

(4) Ecological conservation and fallow cultivation-type land comprehensive consolidation functional area. Including Diaokou Township, Huanghekou Town, Xianhe Town and Xinhu Town, the area is about 2967.24 km^2^, accounting for 35.98% of the total research area. It can be seen from the functional zoning map of land comprehensive consolidation that this area is mainly distributed in the northeast coastal area of Dongying City, with large terrain fluctuations, low population density, and a reclamation rate of about 12%. However, because it is located in the coastal saline–alkali area, it is difficult to cultivate agricultural land. According to research on cultivated land fallow in Peru, long-term fallow can effectively renew soil fertility, biomass and biodiversity, and achieve sustainable use of cultivated land [46]. It is recommended to take fallow and soil management measures to improve soil quality, and to return farmland to areas unsuitable for cultivation, so as to improve the ecological conservation function of the area.

## 4. Conclusions

According to the natural attributes, location attributes, socio-economic attributes and land-use attributes of Dongying City, this study takes the townships as zoning units and uses the entropy method to calculate the weights of each index. Using the grey constellation clustering method, Dongying City is divided into four functional zones of land comprehensive consolidation. Combined with the characteristics and restrictive factors of each sub-region, functional sub-regions for land comprehensive consolidation such as urban development and ecological protection, urban development and cultivated land protection, cultivated land improvement and ecological protection, and ecological conservation and fallow cultivation are formulated. In this way, targeted and precise management of different types of township land resources is carried out, so that land resources can be more fully utilized.

In this study, the grey constellation clustering method was used to divide the research area for land comprehensive consolidation function, and the constellation clustering results were verified by the comprehensive score of the index. This study uses the grey constellation clustering method to carry out the functional zoning of land comprehensive consolidation in the study area. The verification results show that the grey constellation clustering method is suitable for the functional zoning of land comprehensive consolidation in the city area. The functional zoning of land comprehensive consolidation in the study area can provide a classified development direction for land consolidation in Dongying City, provide a basis for the formulation of land consolidation policies, and provide a reference for land consolidation zoning in other areas. However, there is still the problem that the method is not innovative enough in this study, which will be improved in future research.

## Figures and Tables

**Table 1:** Land comprehensive consolidation zoning index system.

Indicator Category	Meta-Metric	Indicator Calculation Formula	Indicator Meaning	Indicator Properties
Natural properties	The proportion of river area	Area of watershed within township/area of each township	Reflects the amount of water contained in each township, and indirectly reflects the irrigation situation of cultivated land.	Positive
	Terrain relief	Extract slope map from DEM	Reflects the topography of each township	Negative
	Forest Vegetation Coverage	Woodland area of each township/area of each township	Reflects the ecological environment construction of each township	Negative
Location attribute	Road network density	Length of roads at all levels in each township/area of each township	Reflects the convenience of road traffic in each township	Positive
	Distance from county center	The distance from the center of each township to the center of the county	Reflects the distance between each township and the county center	Negative
Socioeconomic attributes	Population density	Total population of each township/area of each township	Reflects the degree of intensive and economical use of land in each township	Positive
	GDP per capita	Average annual income of each township/total population of each township	Reflects the socio-economic level of each township	Positive
	Arable land area per capita	Area of arable land in each township/total population of each township	The comparative relationship between population and cultivated land is used to illustrate the scarcity of cultivated land resources	Positive
	Construction land area per capita	Construction land area of each township/total population of each township	Reflects the land-use situation of each township residential area	Positive
Land-use attributes	Intensity of land consolidation	Newly-added cultivated land area of each township/area of each township	Reflects the status quo of the number of agricultural land remediation in each township	Positive
	Proportion of construction land	Construction land area of each township/area of each township	Reflects the overall pattern of land use in each township	Positive
	Reclamation rate	Area of arable land in each township/Area of each township	Reflects the land-use suitability, current situation and development and utilization trend of cultivated land	Positive

**Table 2:** Weights of 12 Meta Indicators.

Indicator Category	Meta-Metric	Weights
Natural properties	The proportion of river area	0.05
	Terrain relief	0.17
	Forest Vegetation Coverage	0.01
Location attribute	Road network density	0.08
	Distance from county center	0.04
Socioeconomic attributes	Population density	0.11
	GDP per capita	0.14
	Arable land area per capita	0.08
	Construction land area per capita	0.09
Land-use attributes	Intensity of land consolidation	0.12
	Proportion of construction land	0.05
	Reclamation rate	0.06

## Data Availability

Not applicable.
